# An unusual case of duodenal neuroendocrine tumor presenting with melena diagnosed by EUS-guided fine-needle biopsy

**DOI:** 10.1097/eus.0000000000000061

**Published:** 2024-06-13

**Authors:** Xianzheng Qin, Taojing Ran, Benyan Zhang, Chunhua Zhou, Duowu Zou

**Affiliations:** 1 Department of Gastroenterology, Ruijin Hospital, School of Medicine, Shanghai Jiao Tong University, Shanghai 200025, China; 2 Department of Pathology, Ruijin Hospital, School of Medicine, Shanghai Jiao Tong University, Shanghai 200025, China.

**Figure 1:**
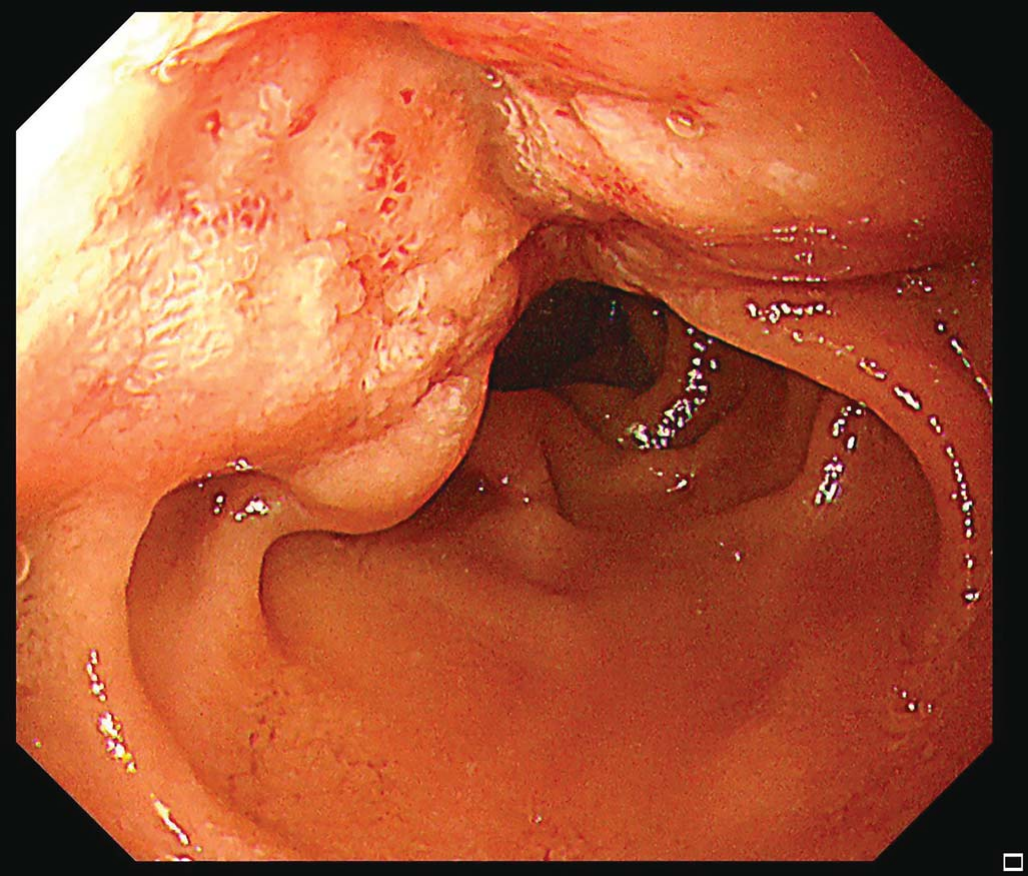
Endoscopic image showed an eminence lesion in the descending part of the duodenum, approximately 30 mm × 30 mm.

**Figure 2:**
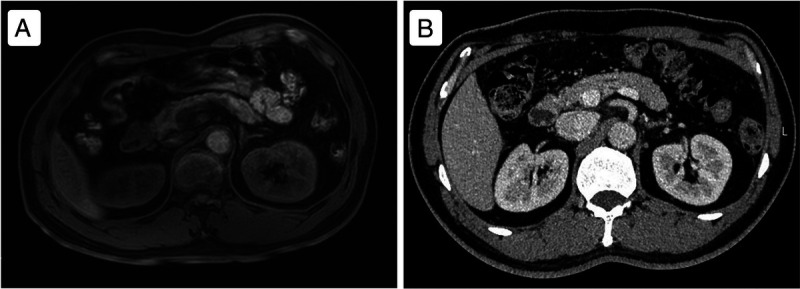
Questionable duodenal lesion. A, Abdominal magnetic resonance imaging revealed duodenal wall thickening, suggestive of angioneurotic edema. B, Enhanced abdominal computed tomography showed the thickening of the duodenal wall and luminal narrowing; the boundary between this affected area and the pancreas was ambiguous.

**Figure 3:**
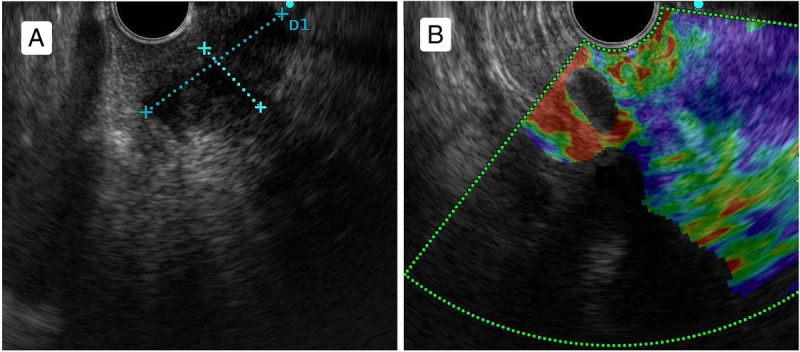
A, EUS showed an inhomogeneous hypoechoic eminence lesion, approximately 26 mm × 21 mm. B, Elastography revealed that the lesion was with hard texture; an elasticity image is expressed as colors (blue: soft, green: average, red: hard).

**Figure 4:**
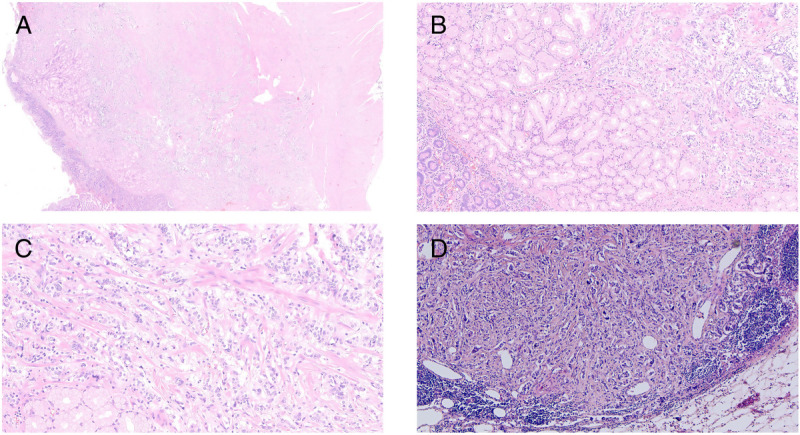
Hematoxylin&Eosin (HE) staining of the surgical specimen. A, B, and C represent HE staining of the duodenal neuroendocrine tumor at different magnifications: (A) at ×40, (B) at ×200, and (C) at ×200; Figure D shows the HE staining of peripancreatic lymph nodes metastases (×200).

**Figure 5:**
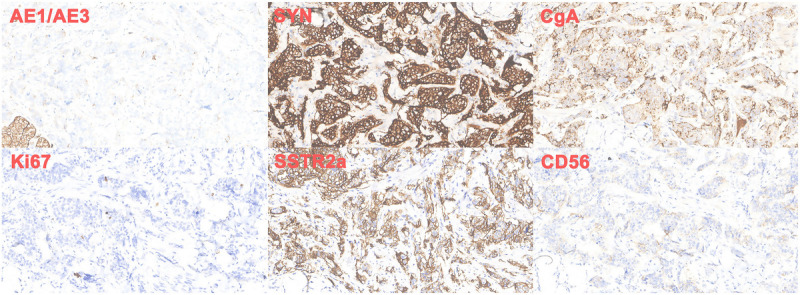
Immunostaining for the surgical specimen. Immunostaining shows positive findings for AE1/AE3, SYN, CgA, SSTR2A, and Ki-67 (index 3%), and negative findings were found in CD56 (×200).

A 56-year-old man presented with recurrent melena for half a month and underwent an upper gastrointestinal endoscopy; an eminence lesion (30 mm × 30 mm) was found in the descending part of the duodenum [Figure 1]. Imaging studies were subsequently conducted. Abdominal magnetic resonance imaging indicated thickening in the descending segment of the duodenum, leading to an imaging diagnosis of angioneurotic edema [Figure 2A]. Contrast-enhanced abdominal computed tomography corroborated the thickening of the duodenal wall and additionally showed luminal narrowing [Figure 2B]. Notably, the boundary between this affected area and a nodular protrusion in the anterior right head of the pancreas was ambiguous. After no significant abnormalities were found in the colonoscopy and capsule endoscopy examinations, the duodenal lesion was considered to be the cause of the upper gastrointestinal bleeding. EUS demonstrated an inhomogeneous hypoechoic eminence lesion in the descending part of the duodenum, measuring approximately 26 mm × 21 mm [Figure 3A], and elastography view showed hard texture of the lesion [Figure 3B]. For more in-depth diagnostic research, a 22-gauge fine-needle biopsy (FNB) needle (Acquire; Boston Scientific, Marlborough, MA) was used to puncture the duodenal lesion during EUS, with the consideration of a duodenal neuroendocrine tumor (DNET). Subsequently, a pancreaticoduodenectomy was performed. Postoperative histopathologic diagnosis suggested DNET, and the tumor cells demonstrate invasive growth, extending upward to the submucosal layer of the duodenum and downward into the extrapancreatic tissue of the intestinal wall, with evidence of neural and vascular invasion [Figure 4]. Immunohistochemical analysis revealed that the tumor cells were diffusely positive for AE1/AE3, SYN, CgA, and SSTR2A, with a Ki-67 index of approximately 3%, whereas CD56 was negative [Figure 5]. Finally, the pathological diagnosis was a DNET of grade 2. A 3-month follow-up showed no complications.

DNETs are rare gastrointestinal tumors and generally have a favorable outcome. Owing to their rarity, DNETs are rarely reported in medical literature. Although DNETs are uncommon, they still account for 20% of upper gastrointestinal bleeding cases that primarily manifest as melena.^[1]^ These tumors tend to originate in the deep mucosa and then spread to the submucosa during the early stages, which leads to them being discovered as subepithelial lesions covered by normal duodenal mucosa during endoscopic screening.^[2]^ Consequently, EUS-FNB should serve as a valuable tool for pathologically diagnosing DNETs, particularly when standard endoscopic methods fail to provide a definitive diagnosis.^[3]^ This is attributed to the capability of EUS-FNB to sample deeper tumor regions and to identify invasive components, which may elude even comprehensive endoscopic forceps biopsies. Therefore, EUS-FNB should be considered a diagnostic option when previous imaging studies or endoscopy indicates the presence of a submucosal or intramural lesion.

In summary, we reported a case of DNET presenting with gastrointestinal bleeding. This study highlights the importance of considering DNET as a differential diagnosis in patients presenting with bleeding, and EUS-FNB plays an important role in the assessment of submucosal duodenal lesions and obtains cytological and histological samples for pathological diagnoses and further helps for determining further strategy in patient treatment.
